# Carbofuran affects cellular autophagy and developmental senescence through the impairment of Nrf2 signalling

**DOI:** 10.1111/jcmm.16774

**Published:** 2021-07-09

**Authors:** Alam Khan, Tanjeena Zaman, Talukdar Mohammad Fahad, Tanjima Akther, Md Faruk Hasan, Tarannum Naz, Shuji Kishi

**Affiliations:** ^1^ Department of Pharmacy University of Rajshahi Rajshahi Bangladesh; ^2^ Department of Molecular Medicine The Scripps Research Institute Jupiter FL USA; ^3^ Department of Fisheries University of Rajshahi Rajshahi Bangladesh; ^4^ Department of Biology University of Hail Hail Kingdom of Saudi Arabia; ^5^ Department of Genetic Engineering and Biotechnology University of Rajshahi Rajshahi Bangladesh; ^6^ S&J Kishi Research Corporation Jupiter FL USA

**Keywords:** autophagy, carbofuran, cellular senescence, Nrf2 pathway, zebrafish

## Abstract

Carbofuran is a broad‐spectrum synthetic pesticide. Its exposure to non‐target mammals affects the biological system through the induction of oxidative stress. Since oxidative stress is a major contributing factor to cellular autophagy and senescence, our present investigation determined the impacts of carbofuran‐induced oxidative stress on cellular autophagy and senescence. A transmembrane protein, Spinster homolog 1 (Spns1), is involved in autophagic lysosomal metabolism. Its mutation accelerates the cellular senescence and shortens the lifespan. Using a transgenic zebrafish line, expressing fluorescent microtubules‐associated protein 1 light chain 3 (EGFP‐LC3) at the membrane of the autophagosome, we found that carbofuran affects autophagic lysosomal biogenesis in wild‐type zebrafish and exacerbates autophagic defect in *spns1*‐mutant zebrafish. In real‐time mortality study, carbofuran has shortened the lifespan of wild‐type fish. Nrf2 is a stress‐responsive transcription factor that regulates the expression of antioxidant genes (such as *gstp1*) in the prevention of oxidative stress‐mediated cellular damage. To assess the effect of carbofuran on Nrf2 signalling, we established a dual‐monitoring transgenic zebrafish line, expressing *gstp1* promoter‐driven EGFP and mCherry‐tagged Neh2 domain of Nrf2. Our results suggested that the exposure of carbofuran has down‐regulated both Nrf2 and Gstp1 expressions. Overall, carbofuran affects cellular autophagy and accelerates senescence by enervating the Nrf2 signalling.

## 1 INTRODUCTION

Senescence is a cellular response towards various stresses, characterized by the permanent proliferative arrest on cells.^1^ Pharmacological and/or genetic ablation of senescence/senescent cells improve health outcomes and extend the lifespan.^2^ Autophagy is a lysosome‐mediated cellular catabolic process, implicated with cellular senescence. In regulating the cellular homeostasis, autophagy usually suppresses cellular senescence by eliminating damaged cytoplasmic organelles and macromolecules.^3^ In contrast, autophagy can also be activated by cellular senescence and up‐regulated in senescent cells.^3^ Under normal physiological conditions, cellular autophagy occurs at low baseline levels, which are up‐regulated under various pathological conditions such as oxidative stress, starvation and insufficient growth factors.^4^ At the beginning of the autophagic process, unnecessary or dysfunctional cytoplasmic constituents form double‐membraned vesicles (known as autophagosomes), which subsequently form autolysosomes via fusion to lysosomes. In autolysosome, the contents of autophagosomes are degraded by lysosomal enzymes and recycled.^5^

In vertebrates, Spinster homolog 1 (Spns1), a lysosomal efflux permease, functions at the terminal stage of autophagy.^6^, ^7^ Mutation of the *spns1* gene in zebrafish (*Danio rerio*) leads to embryonic senescence and premature ageing.^7^, ^8^ Senescence‐associated β‐galactosidase (SA‐β‐gal) is a biomarker to monitor cellular senescence in vitro as well as organismal senescence in vivo.^7^ While higher activity of the lysosomal β‐galactosidase enzyme can be found at lower pH, certainly consistent activity can be detected at pH 6 after treatment with various stressors in zebrafish. Therefore, appropriately conditioned β‐galactosidase activity at pH 6 has been utilized to detect senescent cells as well as to identify genes triggering cellular/embryonic senescence.^9^ In senescent cells, it has also been reported that the accumulation of non‐degradable cellular organelles and macromolecules leads to an increase in lysosomal content.^9^

Carbofuran, chemically known as 2,3‐dihydro‐2,2‐dimethyl‐7‐benzofuranyl methyl‐carbamate, commonly used in agricultural practices to control both leaf‐eating and soil‐dwelling insects.^10^, ^11^ For example, in Bangladesh, about 37% of the total sold pesticides during 2007 was carbofuran.^12^ Carbofuran is non‐specific in its activity, exerts a lethal effect towards a wide range of pests.^12^ Its exposure through contaminated air, water, foods and vegetables may exert noxious effects on non‐target animals including humans.^11^ Carbofuran increases the generation of reactive oxygen species (ROS) in the brain through the inhibition of glutathione‐S‐transferase, catalase and superoxide dismutase.^13^, ^14^ It has also been demonstrated that carbofuran arrests the proliferation of neuronal cells through the induction of oxidative stress.^10^ Excessive ROS generation in the biological system affects cellular fates including apoptosis, autophagy and senescence.^15^ ROS via oxidative stress increase the incidences of age‐related diseases such as atherosclerosis, arthritis and Alzheimer's disease as well as strongly influence the biological ageing process.^2^, ^15^ Recently, we reported that carbofuran hastens the developmental senescence in *spns1*‐mutant zebrafish and affects their lifespan.^16^ The current study extends the report in wild zebrafish, whereas we found carbofuran affects cellular autophagy and accelerates developmental senescence in both wild and *spns1*‐mutant zebrafish.

In the biological system, the oxidative stress condition is due to the imbalance between ROS generation and antioxidative defence mechanisms of the body.^10^ The nuclear factor erythroid 2–related factor 2 (Nrf2) is an essential transcription factor, which promotes the transcription of a number of antioxidant element‐bearing genes.^17^ Under normal physiological condition, Nrf2 remains attached to Kelch‐like ECH‐associated protein 1 (Keap1), which subsequently undergoes proteasomal degradation through ubiquitination. ROS generation usually increases under pathological and stress conditions, where Nrf2 is released from the Keap1‐binding site and translocates into the nucleus. In the nucleus, Nrf2 interacts with the antioxidant response elements (ARE). The interaction promotes the transcriptional activation of antioxidative enzymes such as glutathione‐S‐transferase (GST), catalase and superoxide dismutase. These enzymes detoxify ROS and protect the biological system from oxidative stress.^18^ Therefore, Nrf2 signalling pathways function to maintain cellular homeostasis through the prevention of oxidative stress.^19^, ^20^ Both the induction of oxidative stress and the inhibition of antioxidative enzymes by carbofuran implicate a plausible linkage to the Nrf2 signalling pathway. The detoxifying enzyme pi‐class GST (GSTP1) suppresses cellular oxidative damage by facilitating the conjugation of lethal components to glutathione.^21^ In the present investigation, using dual‐monitoring transgenic zebrafish line that expresses Neh2 domain of Nrf2 as red fluorescence protein (mCherry) and *gstp1* promoter‐driven green fluorescence protein (EGFP), we found carbofuran affects cellular autophagy and developmental senescence by inhibiting Nrf2 signalling.

## 2 MATERIALS AND METHODS

### 2.1 Zebrafish husbandry and maintenance

Adult wild‐type, mutant and transgenic zebrafish were maintained at 25°C in a 14‐h light/10‐h dark cycle. They were fed with brine shrimp once daily. Flake food was also given once daily. A continuously circulating system was used to maintain zebrafish, which replace the water of each tank within 10‐15 minutes of interval. Ultraviolet light was used to disinfect the water of the circulating system. Embryos were raised at 28.5°C until reaching the appropriate stage. Morphological features of embryos and hours of post‐fertilization (hpf) of Kimmel et al (1995) were used to fix the developmental stages of embryos.^22^ Where necessary, 0.003% PTU (1‐phenyl‐2‐thiourea) was used to prevent pigment development.

### 2.2 Chemicals and chemical treatment

Carbofuran was collected from Merck, Germany (SKU 32056). The pesticide was dissolved in DMSO to prepare stock solutions, which diluted with egg water to prepare treatment solutions. The control group treatment solution was prepared by dissolving the same amount of DMSO in egg water. Embryos were treated at different developmental stages for various durations. Longer duration of exposure was used to obtain a persistent effect of carbofuran, whereas old treatment solution was replaced with fresh treatment solution at each 12‐hour interval. Other chemicals, such as 1‐phenyl‐2‐thiourea, paraformaldehyde, potassium ferricyanide, potassium ferrocyanide and MgCl_2_, were also collected from Sigma‐Aldrich, Germany (SKU P7629, 16005, 702587, P3289, and M8266). The Institutional Animal, Medical Ethics, Biosafety and Biosecurity Committee of Institute of Biological Science (I B Sc) of the University of Rajshahi, Bangladesh, has approved zebrafish handling and experimental protocols (protocol id: 245/451/IAMEBBC/IBSc) of the manuscript.

### 2.3 Senescence‐associated β‐gal (SA‐β‐gal) assay

Senescence‐associated beta‐galactosidase (SA‐β‐gal) is a biomarker for senescence induction, commonly used in both cellular and organismal senescence and ageing studies.^23^ Zebrafish embryos of 2 to 5 dpf (days of post‐fertilization) were washed (two times) with phosphate buffer saline (PBS) and fixed in 4% paraformaldehyde (PFA) for at least 12 hours. Afterwards, to completely wash out PFA, the embryos were washed three times in PBS and fourth time washed with staining buffer. Staining buffer consists of 5 mM potassium ferricyanide, 5 mM potassium ferrocyanide and 2 mM MgCl_2_ in PBS at pH 6.0. Then, embryos were incubated at 10°C for 10 hours, via 5‐bromo‐4‐chloro‐3‐indolyl β‐D‐galactopyranoside (X‐gal) solution at a concentration of 20 μg/mL in staining buffer. The chemical compound 5‐bromo‐4‐chloro‐3‐indolyl β‐D‐galactopyranoside interacts with senescence‐associated beta‐galactosidase, which signposted with blue staining. The staining intensity was quantified by pixel analysis, using Adobe Photoshop CS software as described previously.^8^ Due to nearly similar intense blue staining at yolk and yolk extension regions of all embryos, these regions were excluded during the quantification.

### 2.4 Generation of transgenic zebrafish

The pT2‐*gstp1*‐EGFP plasmid containing a 3.5‐kb gene regulatory region of *gstp1* was kindly donated by Dr Kobayashi Lab, Tsukuba, Japan.^24^ In pT2‐Neh2‐mCherry plasmid, mCherry was tagged to Neh2 domain of Nrf2 under the regulation of EF1a (eukaryotic translation elongation factor‐1 α) promoter,^25^, ^26^, ^27^ based on information from literature.^28^, ^29^. To construct Tol2 transposase mRNA, the pCS‐TP plasmid was linearized using digestive enzyme NotI and transcribed by SP6 RNA polymerase kit (Ambion, AM1340). Approximately 2.5 μL pT2‐*gstp1*‐EGFP (200 ng/mL) plasmid or 2.5 μL PT2‐Nrf2‐mCherry (200 ng/mL) plasmid was mixed with Tol2 transposase mRNA, which co‐injected into zebrafish embryos at the one‐cell stage. Embryos having significant expression for both EGFP and mCherry under fluorescence microscope were raised to adulthood and selected germline‐transmitted transgenic founder zebrafish (F0) as previously described.^25^ Positive founders (F0) were in‐crossed to obtain F1 embryos, which were raised to adulthood. Embryos resulting from F1 and/or later generations were used in experiments.

### 2.5 Identification of *spns1^±^* (*hi891*) heterozygous fish

Heterozygous *spns1^±^* (*hi891*) fish was identified using PCR analysis. The PCR primers used to identify *spns1* genomic sequence were forward: 5′‐AGGTAAAGACAGCCCGAAAC‐3′ and reverse: 5′‐GATCCCAGACGCCAACATTA‐3′. The primers used to identify *spns1* (*hi891*) mutant sequence were forward: 5′‐TAAGTCGGTCGGCTGCACGGTT‐3′ and reverse: 5′‐TGATCTCGAGTTCCTTGGGAGGGTCT‐3′. Heterozygous *spns1^±^* (*hi891*) fish should have both *spns1* genomic and *spns1* (*hi891*) mutant sequences. Homozygous embryos are harbouring only *spns1* (*hi891*)‐mutant sequences and lack of *spns1* genomic sequence. Homozygous *spns1* ^−/−^ embryos were also recognized by morphological phenotypes such as yolk opacity around 1‐2 dpf, loss of yolk extension at 3‐4 dpf and death around 4‐5 dpf.^7^, ^8^

### 2.6 Survival analysis

Homozygous *spns1* ^−/−^ embryos were sorted based on the yolk opaqueness phenotype and exposed to carbofuran (100 µM) from 36 hpf to until the death of all embryos. Embryos were monitored and counted under a stereo‐microscope at each 6‐hour interval. Whenever a dead embryo was observed, it has been removed from the treatment preparation. In the case of real‐time mortality study, adult zebrafish of wild‐type background has been continuously exposed to a low dose of carbofuran (5 µM) through fish water, since 3 months of post‐fertilization. Survival data were presented through the Kaplan‐Meier survival curve.

### 2.7 LysoTracker red staining

EGFP‐tagged microtubules‐associated protein 1 light chain 3 (EGFP‐LC3) expresses at the membrane of autophagosomes, which is widely used as a biomarker to estimate the progress in the autophagic process.^30^, ^31^ In our estimation, we used a transgenic zebrafish of *spns1^±^* (*hi891*) heterozygous background (Tg[EGFP‐LC3]; *spns1* ^±^), expressing EGFP‐LC3 under the control of CMV (human cytomegalovirus) promoter.^30^ Transgenic zebrafish embryos of homozygous background (Tg[EGFP‐LC3]; *spns1* ^−/−^) were obtained via in‐cross among adult transgenic zebrafish of heterozygous background. The lysosomal dye, LysoTracker Red DND‐99 (Invitrogen/Molecular Probes, L7528), is a biomarker for lysosome. The dye (1 μM, 1 μL) was added to 200 μL E3 medium (0.33 mM CaCl2, 0.33 mM MgSO4, 0.17 mM KCl, 5 mM NaCl) consisting of around 20 embryos and incubated in the dark condition at 28.5°C. After incubation for around one hour, the embryos were washed three times with E3 medium and subjected to image capturing.

### 2.8 RT‐PCR analysis

The *gstp*1 mRNA expression level was estimated using RT‐PCR analysis. Total RNA from the embryos of each group was isolated using TRIzol reagent (Invitrogen), considering manufacturer's instruction. Total RNA was reverse transcribed in cDNA using M‐MLV reverse transcriptase (Promega). As a control, the expression level of the *β‐actin* mRNA was estimated. RT‐PCR primers used to amplify the fragments of genes were designed using PrimerQuest website (Integrated DNA Technology). The primers used were *gstp1* forward: 5′‐CTAGGAGCAGCTTTGAAACGCAC‐3′, *gstp1* reverse: 5′‐CGTTGTTGGAGAATGTTGTACCGACG‐3′, *β‐acti*n forward: 5′‐CCCAGACATCAGGGAGTGAT‐3′, and *β‐actin* reverse: 5′‐CACCGATCCAGACGGAGTAT‐3′.

### 2.9 Microscopy and imaging

Before imaging, embryos were anaesthetized using 0.16 mg/mL tricaine (Sigma, A5040). Imaging was carried out with FluoView 1000 confocal microscope system (Olympus). Embryos subjected to SA‐β‐gal experiments were photographed under reflected light of the stereo‐microscope (Opto‐Edu A23.1002‐B). Fluorescence images of whole embryos were captured using fluorescence light of the macro‐fluorescence microscope (Nikon AZ100). For each particular determination, all images were photographed under the same condition. Fluorescence intensities of captured images were quantified using Adobe Photoshop software.

### 2.10 Statistical analysis

Statistical analyses were carried out using Statistical Package for the Social Sciences (SPSS) software version 20.0. The software was used to generate each of the graphs. Data were expressed as mean ± SE. Comparisons between different animal groups were made with Student's *t* test.

## 3 RESULTS

### 3.1 Carbofuran accelerates developmental senescence and affects biological ageing

**FIGURE 1:**
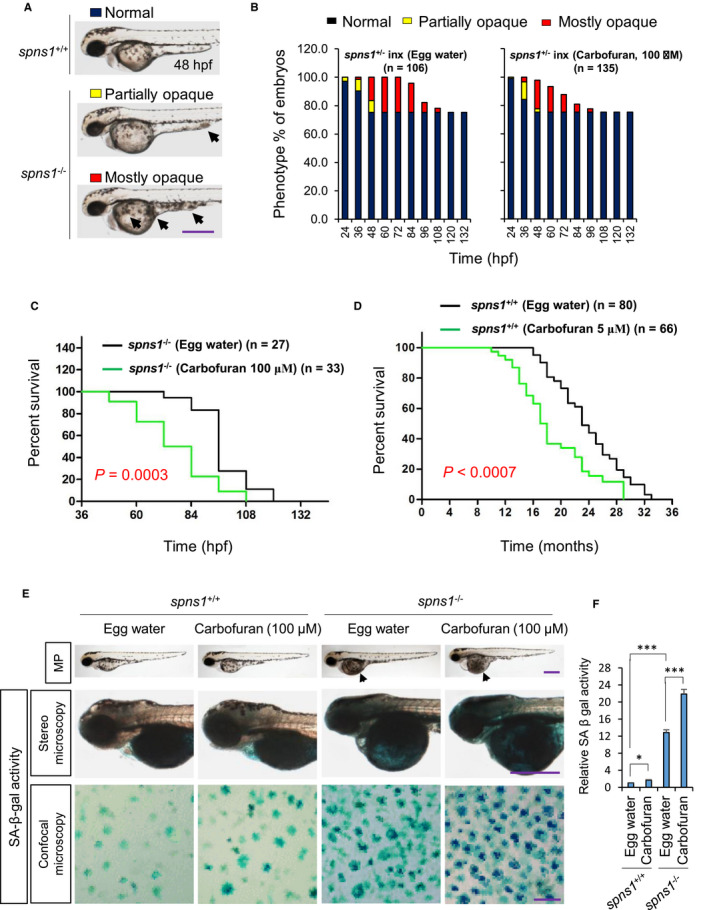
Carbofuran exacerbates cellular senescence and shortens lifespan. (A) Based on the magnitude of opaqueness, the yolk opaqueness was sorted as partially opaque (marked by yellow colour) and mostly opaque (marked by red colour). Fish (wild type; *spns1* ^+/+^ or heterozygote; *spns1* ^±^) did not show opaqueness phenotype was marked by black colour. (B) The effect of carbofuran exposure on senescence phenotype (yolk opacity) of *spns1*‐mutant zebrafish. In the cases of carbofuran‐treated *spns1*‐mutant zebrafish, both partially and mostly opaque phenotypes were found at earlier times than mutant zebrafish of the control group (egg water treatment). In addition, carbofuran treatment leads mutant zebrafish to die at quicker times than fish of the control group. (C) Survival (in hours) of *spns1*‐mutant embryos has been shortened (*P* = .0003) upon exposure to carbofuran. (D) Survival (in months) of wild zebrafish was shortened (*P* < .0007) upon exposure to carbofuran. (E) Carbofuran accelerates SA‐β‐gal activity in both wild‐type (*spns1* ^+/+^) and *spns1*‐mutant (*spns1* ^−/−^) zebrafish. Carbofuran did not affect morphological phenotype (MP) of wild fish but affected yolk opaqueness of *spns1*‐mutant fish (black arrowhead in the first row). SA‐β‐gal–stained fish have shown in the second row. The cellular SA‐β‐gal staining at dorsal to the eye has shown in the third row. (F) Quantification of dark blue particles of SA‐β‐gal staining of whole fish. The scale bar is 250 μm (stereo microscopic images) and 10 μm (confocal microscopic images). The number of fish was 10 (n = 10). Data are presented as mean ± SE. *P ≤ .05; ***P ≤ .005

Embryos, lacking the *spns1* gene, can be morphologically identified by yolk opacity, smaller eye and loss of yolk extension.^7^, ^8^ Such embryos cannot survive, but die within 3‐6 days of post‐fertilization. We used heterozygous *spns1* ^±^ fish of males and females for cross‐mating. Among resultant embryos, homozygous (*spns1* ^−/−^) mutant embryos were identified by the representative phenotypes such as yolk opacity (Figure 1A). It was estimated that about 25% of resultant embryos were developed with the yolk‐opaque phenotype, due to Spns1 deficiency.

Of note, heterozygous *spns1* ^±^ fish, as well as normal fish, do not show the yolk‐opaque phenotype,^7^ and in almost all cases, yolk opaqueness of homozygous *spns1* ^−/−^ fish originated at the ventral tip of yolk extension and gradually enlarged towards other parts of yolk extension and yolk (Figure 1A). Based on the magnitudes of opaqueness, the yolk opacities were classified as partially opaque and mostly opaque (Figure 1A). Carbofuran exposure to *spns1*‐mutant zebrafish induced yolk‐opaque phenotypes at earlier times than *spns1*‐mutant fish of the control group. Moreover, carbofuran‐treated mostly opaque fish have died faster than the fish of the control group (Figure 1B).

The loss of the *spns1* gene induces organismal and cellular senescence, which can be determined by the robust up‐regulation of SA‐β‐gal activity.^8^ The strong increment of SA‐β‐gal activity in *spns1*‐mutant zebrafish has also been confirmed in our current study (Figure 1E, F). Further, we found that carbofuran exposure to wild‐type zebrafish has increased SA‐β‐gal activity. Although the increment was relatively weak, nevertheless the activity was significantly increased, suggesting carbofuran enhances senescence in zebrafish embryos.

In survival analysis, we reconfirmed that carbofuran exposure significantly shortened the lifespan of *spns1*‐mutant zebrafish (Figure 1E).^16^ Additionally, using a real‐time survival study, we found carbofuran significantly abated the survival lengths of wild zebrafish (Figure 1F), suggesting carbofuran affects lifespan of both wild and *spns1* ^−/−^ mutant fishes.

### 3.2 Carbofuran affects autophagy process in both wild and *spns1*‐mutant fishes

**FIGURE 2:**
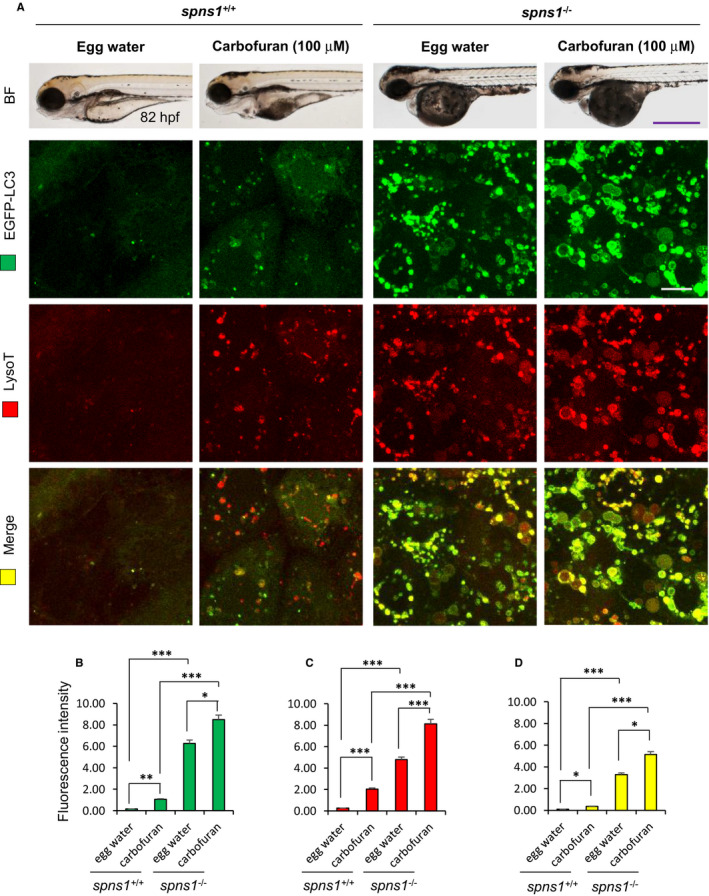
Carbofuran affects autophagy through autolysosomal protein accumulation. (A) Carbofuran affects autophagy in both wild‐type and *spns1*‐mutant fish. The top panel is the observation under the bright field condition of the stereo‐microscope. The second (EGFP‐LC3) and third (LysoTracker Red) panels were observed under fluorescence conditions of the confocal microscope (head region, dorsal to the eye). Merged images (yellow) have shown in the bottom panel. (B) Alteration of autophagosomal expression by carbofuran treatment. In both wild and *spns1*‐mutant zebrafish, carbofuran treatment significantly increased autophagosomal EGFP‐LC3 expression. (C) Alteration in lysosomal expression by carbofuran treatment. In both wild and *spns1*‐mutant zebrafish, carbofuran treatment significantly increased lysosomal LysoTracker Red expression. (D) Co‐localization of autophagosomal EGFP and lysosomal LysoTracker Red expressions. Up‐regulated autophagosomal and lysosomal expressions are significantly colocalized. Scale bar: 250 μm (stereo macro‐microscopic images) and 10 μm (confocal microscopic images). The number of animals was 6 (n = 6). Data are presented as mean ± SE. *P ≤ .05; ***P ≤ .005

The loss of *spns1* function causes the accumulation of autolysosome in the cell, which was recognized through the accumulation of enlarged lysosomal and autophagosomal positive structures as well as their co‐expression.^6^ Furthermore, in our previous study, it has been demonstrated that Spns1 plays a pivotal role in the regulation of cellular autophagy in zebrafish.^31^ Thus, carbofuran was exposed to transgenic zebrafish of *spns1*‐mutant background, expressing EGFP‐LC3 at the membrane of autophagosome (Tg[EGFP‐LC3]; *spns1* ^−/−^), and subjected to LysoTracker Red staining. Carbofuran exposure to *spns1*‐mutant zebrafish has been enhanced both autophagosomal and lysosomal puncta and their accumulation (head region, dorsal to the eye) (Figure 2A‐C). The enhanced autophagosomal and lysosomal signals were significantly colocalized (Figure 2A,D), suggesting carbofuran exposure has further deteriorated the autophagic defect in *spns1*‐mutant zebrafish. In cells with normal physiological conditions, autophagy still occurs at baseline magnitudes, which usually up‐regulated under pathological conditions including stressful environments^32^ Likewise in *spns1*‐mutant fish, both autophagosomal LC3 (green) expression and lysosomal (red) staining signals were up‐regulated by carbofuran exposure to wild‐type zebrafish (Figure 2A‐C). While the increased signal pattern in the wild‐type fish treated with carbofuran was still relatively weaker than the signal in *spns1*‐mutant zebrafish, it was significantly increased in comparison with the signal of the control group (Figure 2B,C). Co‐localization of lysosomal and autophagosomal signal in carbofuran‐treated zebrafish was also significantly increased (Figure 2D). These data suggest that carbofuran itself transiently induces autolysosomal formation and accumulation (autolysosomal biogenesis), and cooperatively functions with Spns1.

### 3.3 Carbofuran interferes Nrf2‐ARE transcriptional regulatory elements

**FIGURE 3:**
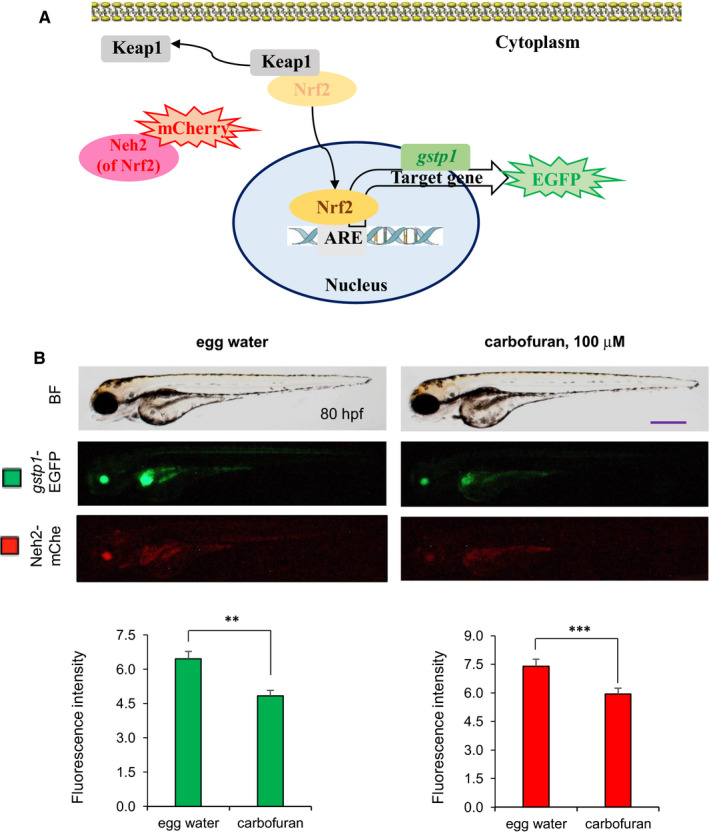
Carbofuran affects Nrf2 signalling. (A) Schematic illustration of the Nrf2‐ARE signalling pathway as well as monitoring the pathway using a transgenic model. Nrf2 translocates into the nucleus by releasing Keap1. In the nucleus, Nrf2 transactivates target genes including *gstp1* through binding to ARE regulatory element. The Nrf2‐ARE signalling pathway was monitored using a double‐transgenic zebrafish line expressing Neh2 domain (of Nrf2)‐mCherry and *gstp1*‐EGFP. (B) Effect of carbofuran on the Nrf2‐ARE signalling pathway. Embryos were treated with carbofuran for 24 hours, started from 56 hpf and observed under both bright field (BF) (top row) and fluorescence conditions (bottom two rows) of the microscope. Both Neh2‐mCherry and *gstp1*‐EGFP expressions were down‐regulated by carbofuran exposure. Scale bar: 250 μm. The number of animals was 10 (n = 10). Data are presented as mean ± SE. **P ≤ .01; ***P ≤ .005

The stress‐ or chemical‐mediated activation of the Nrf2 pathway displaces Nrf2 component from the Keap1‐binding site to the nucleus, where it binds to ARE and regulates the expression of genes of the defence system including *gstp1*.^33^ Therefore, we established a dual‐monitoring transgenic zebrafish line, which enables us to visualize and monitor both mCherry‐tagged Neh2 (a domain portion of Nrf2) and *gstp1*‐driven EGFP expressions (Figure 3A). The transgenic zebrafish embryos were exposed to carbofuran through egg water since 56 hpf and monitored under a fluorescence microscope. Carbofuran treatment did not affect embryos morphologically; however, both mCherry‐Neh2 and *gstp1*‐EGFP expressions were significantly declined by 80 hpf, suggesting carbofuran impairs cellular Nrf2 defence pathway (Figure 3B‐D).

### 3.4 Differential effects of carbofuran on Nrf2 signalling are independent of its effect on autophagy progress

**FIGURE 4:**
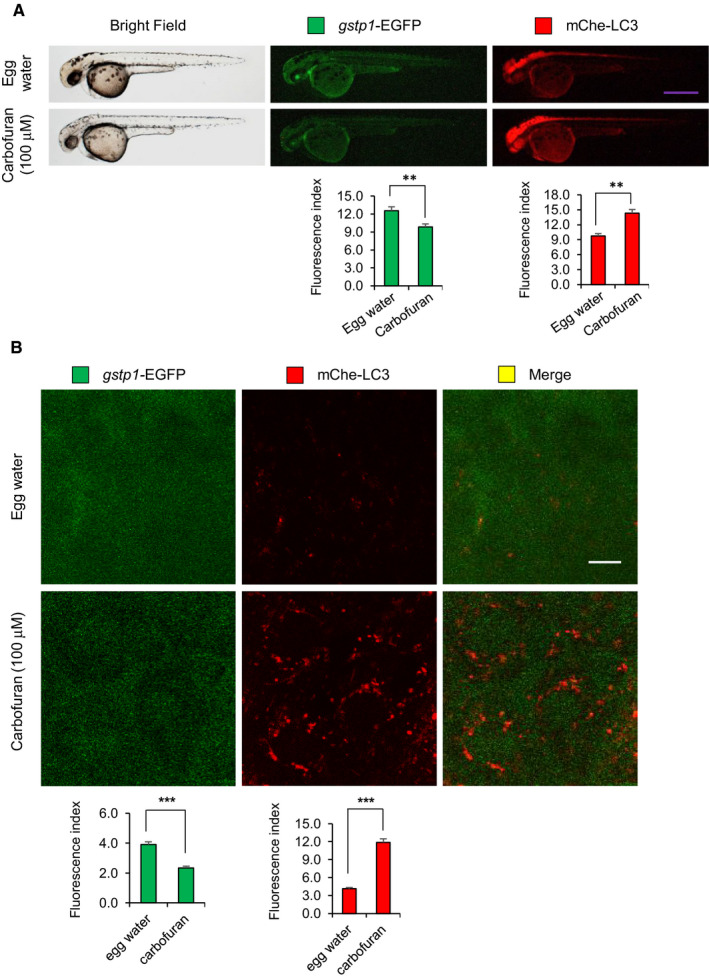
Carbofuran down‐regulates Gstp1 (This is fine, no change!) expression and up‐regulates autophagosomal LC3 expression. (A) The gross expressions of *gstp1*‐EGFP and mCherry‐LC3 in transgenic zebrafish under macro‐microscope. Transgenic zebrafish were exposed to carbofuran for 12 hours (48 to 60 hpf). Carbofuran treatment significantly declined *gstp1*‐EGFP expression and significantly increased autophagosomal mCherry‐LC3 expression throughout the fish. Scale bar: 250 μm. (B) Cellular *gstp1*‐EGFP and mCherry‐LC3 expressions (head region, dorsal to the eye) under the confocal microscope. Like gross observation, carbofuran treatment significantly declined cellular *gstp1*‐EGFP expression and significantly increased autophagosomal mCherry‐LC3 expression. Scale bar: 10 μm. The number of animals was 8 (n = 8). Data are presented as mean ± SE. **P ≤ .01; ***P ≤ .005

**FIGURE 5:**
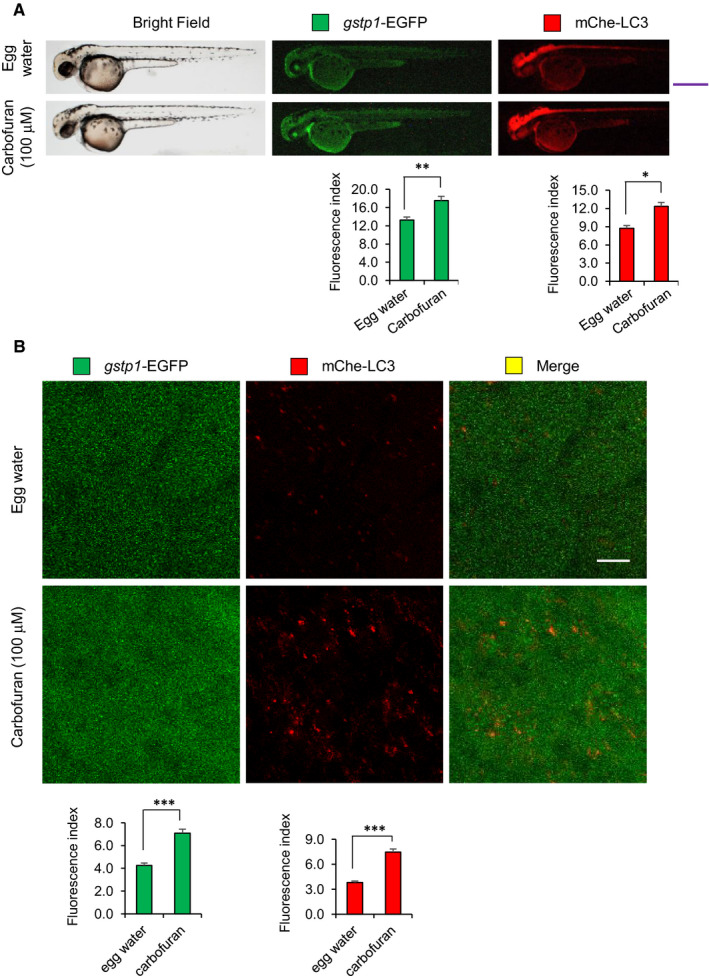
Carbofuran exposure for a short duration of time up‐regulates both Gstp1 (This is fine, no change!) and autophagosomal LC3 expressions. (A) The gross expressions of *gstp1*‐EGFP and mCherry‐LC3 in transgenic zebrafish. Carbofuran treatment for a short duration of time (56 to 60 hpf) significantly increased both *gstp1*‐EGFP and autophagosomal mCherry‐LC3 expressions throughout the fish. Scale bar: 250 μm. (B) Cellular *gstp1*‐GFP and mCherry‐LC3 expressions (head region, dorsal to the eye) under the confocal microscope. Like gross observation, carbofuran treatment significantly increased both cellular *gstp*1‐EGFP and autophagosomal mCherry‐LC3 expressions. Scale bar: 10 μm. The number of animals was 8 (n = 8). Data are presented as mean ± SE. *P ≤ .05; **P ≤.01; ***P ≤ .005

**FIGURE 6:**
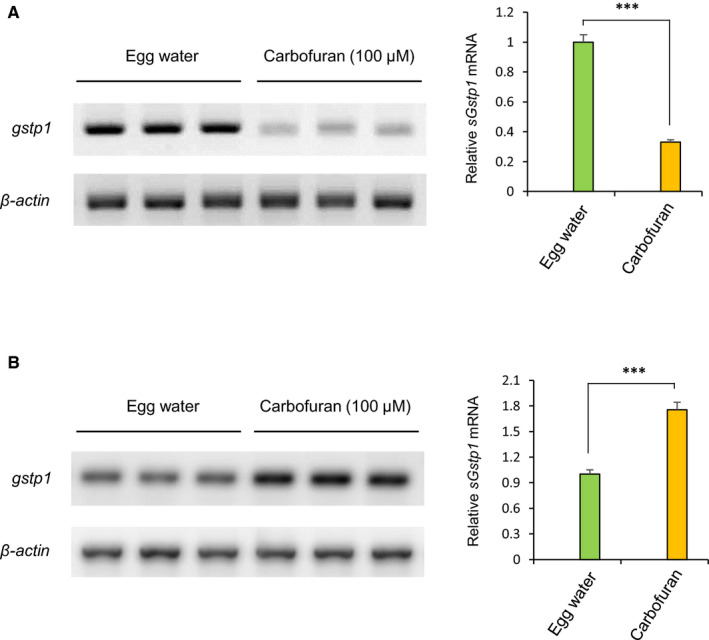
Differential regulation of *gstp1* mRNA expression based on the duration of carbofuran exposure. (A) Persistent carbofuran exposure (12 hours) down‐regulates the *gstp1* mRNA level. B, Carbofuran exposure for a short duration of time (4 hours) up‐regulates the *gstp1* mRNA level. Data are mean ± SE (n = 3 samples, 3 embryos per sample). ***P ≤ .005

Under oxidative stress, ROS generated in mitochondria are essential mediators of cellular autophagy.^34^ It has been demonstrated that carbofuran induces oxidative stress in mammals through the up‐regulation of ROS generation.^14^ To investigate the impact of carbofuran‐induced oxidative stress on autophagy progress, a double‐transgenic zebrafish line, expressing *gstp1*‐driven EGFP and mCherry‐tagged LC3 (Tg [*gstp1*‐EGFP;mCherry‐LC3]), has been used. Adult transgenic zebrafish were in‐crossed, and the resulting embryos were exposed to carbofuran for 12 hours (long‐term exposure), started from 48 hpf (by the endpoint of 60 hpf) during embryonic development. Embryos were examined and observed under a stereo‐fluorescence microscope to monitor the fluorescent protein expression throughout the body as well as under a confocal microscope to monitor the expression at the cellular level. The expression of *gstp1*‐EGFP was declined, whereas the expression of mCherry‐LC3 was up‐regulated throughout the body (Figure 4A). A similar pattern of changes in expressions was observed at the cellular level (Figure 4B), where an accumulation of autophagosomal mCherry‐LC3 puncta was observed (as a hallmark of autophagy induction). On the other hand, embryos exposed to carbofuran for a short duration (4 hours; short‐term exposure) from 56 hpf (by the endpoint of 60 hpf) showed an opposite pattern of expression for *gstp1*‐EGFP; that is the up‐regulation of the potential Gstp1 activity (Figure 5A,B). However, autophagosomal LC3 expression was still increased after the short duration of carbofuran exposure likewise the long duration of carbofuran exposure. The distinction in Gstp1 activity upon long and short intervals of carbofuran exposure was further confirmed by RT‐PCR analysis (Figure 6A,B). Overall, Gstp1 activity was initially up‐regulated by carbofuran exposure, but subsequently down‐regulated upon persistent continuous exposure, whereas carbofuran‐induced autophagosomal puncta seemed constitutive and were observed under both long and short terms of carbofuran exposure.

## 4 DISCUSSION

**FIGURE 7:**
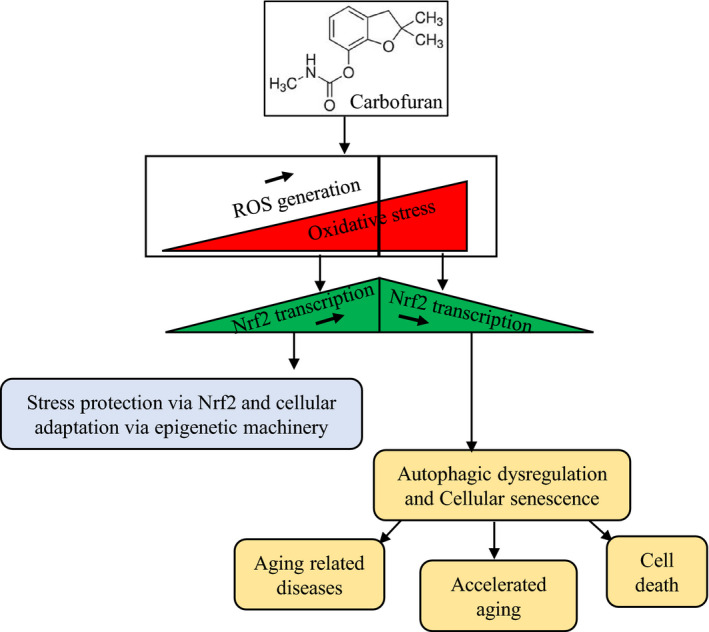
A proposed schematic model of carbofuran induced declines in biological ageing through impairment of Nrf2 signalling. Carbofuran exposure induces oxidative stress through ROS generation. At low to the medium stress conditions, cells protect themselves via Nrf2‐mediated transactivation of antioxidant regulatory genes as well as through epigenetic modification. Persistent carbofuran exposure increases oxidative stress, under which the cellular protective mechanism falls and impair the Nrf2 signalling. Ultimately, the suppression of the Nrf2 signalling induces cellular senescence and affects biological ageing

Among carbamate pesticides, carbofuran is one of the most widely used pesticides in agriculture and forestry, due to its noxious effects against a large number of insects.^35^ Its environmental exposure to humans leads to toxic effects such as spermatozoal DNA damage and retinal degeneration.^36^ Studies with rats have suggested that carbofuran induces oxidative stress in the nervous system and skeletal muscle by disrupting pro‐oxidant/antioxidant balance.^37^ Carbofuran also induces oxidative stress in the liver through increasing lipid peroxidation and decreasing hepatic superoxide dismutase.^38^ A basal level of ROS generation is required for cell survival.^39^ At low to the moderate extent of oxidative stresses, cellular protection against oxidative stress is carried out by Nrf2‐mediated transactivation of ARE‐targeted genes as well as using cellular adaptation via epigenetic modification such as DNA methylation and/or histone modification (Figure 7).^39^ Persistent oxidative stress may cause cellular senescence.^40^ In the present investigation, we revealed carbofuran up‐regulates the cellular senescence in both wild and *spns1*‐mutant zebrafish in accordance with dysregulation of stress response and autophagy. It has been demonstrated that the *spns1* deficiency induces cellular senescence and shorten lifespans in both fruit fly and zebrafish.^41^ Although carbofuran significantly increased senescence in wild‐type zebrafish, the acceleration of senescence effect in *spns1*‐mutant zebrafish was more prominent, demonstrating that the carbofuran‐induced stress had an additive impact in *spns1*‐mutant zebrafish, and hence synergistically accelerated the senescence phenotype.

Cellular senescence is a potent mediator of cell cycle arrest; however, the cell cycle may also be blocked by other factors. For example, withdrawal of growth factors and nutrients from serum can cause cell cycle arrest.^42^ Fu et al (2019)^43^ consistently found the implication of carbofuran in cell cycle arrest and cellular apoptosis. Acute senescence may be a part of normal physiological processes, which are beneficial for balanced embryonic development and wound healing, whereas chronic senescence (usually referred as senescence) is a hallmark of ageing, which leads to irreversible cell cycle arrest.^44^, ^45^ Senescence is associated with age‐related diseases, such as neurodegenerative disorders, cancer, diabetes and cardiovascular diseases, and subsequently declines biological lifespan.^44^, ^46^ Induction of cellular senescence in *spns1*‐mutant fish is linked to premature ageing phenotype in adults and their shortened lifespan^7^, ^8^ In addition to the acceleration of senescence phenotype, carbofuran exposure to *spsn1*‐mutant fish has exacerbated the yolk opaqueness phenotype as well as shortened the lifespan of *spsn1*‐mutant fish. The lifespan of wild‐type zebrafish has also shortened upon carbofuran exposure. The decline in life expectancy by carbofuran treatment might be due to senescence‐induced cell cycle arrest. Overall, carbofuran accelerates cellular senescence and affects life expectancy in both wild‐type and *spsn1*‐mutant fishes.

Oxidative stress through the generation of ROS is a function of the major intracellular signal transduction in autophagy. ROS, particularly O_2_^−^ and H_2_O_2_, are among the main signalling molecules required for autophagy execution.^47^, ^48^ Mitochondria as a principal site of ROS generation can commence autophagy.^47^ It has been reported that carbofuran increases ROS and free‐radical generation via the impairment of mitochondrial electron transport chain.^13^ The sub‐chronic carbofuran exposure increases around 44% of mitochondrial lipid peroxidation. Carbofuran induces an impairment of the electron transport chain in the mitochondria,^13^ which might be a stimulating factor in the autophagy process. Autophagy progress was determined by autophagosomal and lysosomal biogenesis, which were up‐regulated by carbofuran exposure to both wild‐type and *spns1‐*mutant zebrafish. As autophagy can also be triggered by cellular senescence,^3^ therefore, acceleration of senescence by carbofuran exposure may additionally contribute to the increment in autophagosomal and lysosomal biogenesis. The size of both autophagosomal and lysosomal puncta was increased by carbofuran exposure, and those puncta were sustainably colocalized, which suggests an autolysosomal accumulation, due to their improper biogenesis.

The activation of Nrf2 signalling is crucial for cellular adaptive and protective responses to oxidative stress.^49^ Several chemicals of both natural (eg sulforaphane, arsenic) and synthetic (eg oltipraz, paraquat) sources transactivate ARE genes via the Nrf2 pathway.^50^ Conversely, chemicals of both natural (eg apigenin, brusatol) and synthetic (eg isoniazid, aminopyrazine) sources suppress Nrf2 signalling.^49^ Carbofuran exposure to zebrafish has affected Nrf2 signalling depending on the duration of exposure. Exposure for a short duration (4 hours) found activates Nrf2 pathway, whereas sustained exposure (12 hours or more) suppressed Nrf2 signalling. The initial activation of Nrf2 signalling might be due to an increase in ROS generation by carbofuran exposure. The cysteine residue of Keap1 is oxidized by ROS, which results in the activation of Nrf2.^51^ On the other hand, the suppression of Nrf2 signalling upon persistent carbofuran exposure might be suggested according to carbofuran‐induced senescence and ageing disorders. Under ageing‐related pathological circumstances such as diabetes and Chagas disease, ROS impairs Nrf2 signalling.^52^, ^53^ Furthermore, the transcriptional activity of Nrf2 declines with ageing and allied pathologies such as atherosclerosis, Alzheimer's and Parkinson's disease.^54^ In elderly people, circulating insulin‐like growth factor‐1 (IGF‐1) is down‐regulated, which leads to age‐associated vascular diseases, including atherosclerosis.^55^ In mice, it was demonstrated that reduced circulating IGF‐1 declines the Nrf2 signalling.^56^ In Alzheimer's (AD) and Parkinson's diseases, the translocation of Nrf2 into the nucleus is impaired, which decreases cellular protection under oxidative stress.^57^ Cellular senescence and ageing are implicated with each other. A rapamycin‐insensitive protein complex, mTORC2, facilitates senescence in vascular endothelial cells by reducing Nrf2 transcription.^58^ The impairment may also be associated with the neurodegenerative effect of carbofuran as neuronal degeneration inhibits Nrf2 translocation into the nucleus.^57^

Since both short and persistent exposure conditions of carbofuran up‐regulated autophagosomal LC3 expression, initial up‐regulation is due to carbofuran‐induced ROS generation, and subsequent up‐regulation might be because of carbofuran‐induced cellular senescence and neurodegeneration. All these conditions (ROS generation, cellular senescence and neurodegeneration) are effective to stimulate autophagosome formation.^3^, ^47^, ^59^

While we found an initial phase of Nrf2 activation by carbofuran, overall, our data suggested that carbofuran persuades impaired autolysosomal accumulation as well as accelerates senescence via subsequently possible dysfunction of the Nrf2 pathway. However, we still need further investigation on the mechanism underlying the role of carbofuran that affects the Nrf2 pathway by linking to Spns1.

## CONFLICT OF INTEREST

The authors have no competing interest.

## AUTHOR CONTRIBUTIONS

**Alam Khan:** Conceptualization (lead); Investigation (lead); Project administration (lead); Writing‐review & editing (lead). **Tanjeena Zaman:** Conceptualization (equal); Formal analysis (equal); Investigation (equal); Writing‐original draft (equal); Writing‐review & editing (equal). **T M Fahad:** Investigation (equal); Writing‐review & editing (equal). **Tanjima Akther:** Investigation (equal); Writing‐review & editing (equal). **Md Faruk Hasan:** Investigation (equal); Writing‐review & editing (equal). **Tarannum Naz:** Investigation (equal); Writing‐review & editing (equal). **Shuji Kishi:** Conceptualization (equal); Writing‐original draft (equal).

## Data Availability

The data that support the finding of this article are available from the corresponding author upon reasonable request.
